# Senescence-Related Changes in Gene Expression of Peripheral Blood Mononuclear Cells from Octo/Nonagenarians Compared to Their Offspring

**DOI:** 10.1155/2013/189129

**Published:** 2013-11-27

**Authors:** Amirah Abdul Rahman, Norwahidah Abdul Karim, Noor Aini Abdul Hamid, Roslan Harun, Wan Zurinah Wan Ngah

**Affiliations:** ^1^Department of Biochemistry, Faculty of Medicine, Universiti Kebangsaan Malaysia, Jalan Raja Muda Aziz, 50300 Kuala Lumpur, Malaysia; ^2^Cyberjaya University College of Medical Sciences, 63000 Cyberjaya, Selangor, Malaysia; ^3^UKM Medical Molecular Biology Institute (UMBI), UKM Hospital, Level 7, Clinical Block, Cheras, 56000 Kuala Lumpur, Malaysia

## Abstract

Mechanisms determining both functional rate of decline and the time of onset in aging remain elusive. Studies of the aging process especially those involving the comparison of long-lived individuals and young controls are fairly limited. Therefore, this research aims to determine the differential gene expression profile in related individuals from villages in Pahang, Malaysia. Genome-wide microarray analysis of 18 samples of peripheral blood mononuclear cells (PBMCs) from two groups: octo/nonagenarians (80–99 years old) and their offspring (50.2  ±  4.0 years old) revealed that 477 transcripts were age-induced and 335 transcripts were age-repressed with fold changes ≥1.2 in octo/nonagenarians compared to offspring. Interestingly, changes in gene expression were associated with increased capacity for apoptosis (*BAK1*), cell cycle regulation (*CDKN1B*), metabolic process (*LRPAP1*), insulin action (*IGF2R*), and increased immune and inflammatory response (*IL27RA*), whereas response to stress (*HSPA8*), damage stimulus (*XRCC6*), and chromatin remodelling (*TINF2*) pathways were downregulated in octo/nonagenarians. These results suggested that systemic telomere maintenance, metabolism, cell signalling, and redox regulation may be important for individuals to maintain their healthy state with advancing age and that these processes play an important role in the determination of the healthy life-span.

## 1. Introduction

The aging process determined by genetic and environmental factors remains unchanged despite increasing the average life-span of the general population in recent decades [1]. The heritability component of human longevity ranged from 20% to 30% and can increase to about 50% after the age of 60 [2]. Thus, the search for genes affecting longevity in humans was mostly conducted on subjects at an advanced age involving centenarians. Families living such extremely long lives perhaps possess genetic variations that affect either the rate of aging or genes that result in decreased susceptibility to age-associated diseases [1]. Identification of innate genes that changes in expression with age, in a small population to minimize variations arising from environmental factors such as diet and lifestyle, can be useful as targets for intervention or can be used as biomarkers of aging.

Uncovering interactions of major regulatory pathways and targets is crucial to elucidate aging mechanisms. Some of the most promising candidate genes appear to be involved in regulatory pathways such as stress resistance, immune/inflammatory response, insulin signalling, or cardiovascular function. Such information could be extracted from transcriptomic studies [3]. However, reports on gene expression analysis of human aging are usually focused on determining small age changes and were based on candidate genes. Global changes in gene expression were investigated mainly as comparison between diseased and healthy subjects, while changes in mRNA expression patterns with age in humans are limited. Moreover, compared to genetics studies, the relation in gene expression profiles associated with life-span and longevity is less emphasized [4]. To this end, studies looking into longevity genes usually involved long-lived individuals and younger controls that are not related [1]. A comparison of long-lived individuals and their offspring could provide some insights of differentially expressed innate genes and provide insight into time dependent changes in expression of certain genes.

Various tissues across model organisms such as brain [5] were studied in relation to aging. Yet, the effect of aging is not limited to one specific tissue. The use of peripheral blood mononuclear cells (PBMC), which has been accepted to represent changes in the whole body, may provide a useful tool to study human aging [4]. It is suggested that PBMC may also represent the biological processes occurring in the body relevant to aging and longevity. Processes affecting the body system may be reflected in the blood where blood parameters have been successfully used for classification of disease subgroups and disease progression rates and monitoring of medical treatment [4]. Therefore, this study was conducted to determine the differential expression of human PBMC in healthy octo/nonagenarians in comparison with their offspring.

## 2. Experimental Procedures

### 2.1. Recruitment of Subjects

This study was approved by the Ethics Committee of Universiti Kebangsaan Malaysia (UKM). After obtaining written informed consent, recruitment of subjects with comparable lifestyle and diet was conducted at villages located in Pahang, Malaysia, to control for environmental and lifestyle factors. A total of 40 subjects were prescreened and 18 subjects of healthy octo/nonagenarians and offspring of octo/nonagenarians that best fit the inclusion criteria were included in the study. The age of the offspring volunteers ranged between 45 and 56 years old. As for octo/nonagenarians, participants recruited fulfilled the inclusion criteria of age between 80 and 99 years, not institutionalized, and have no evidence of medical conditions or chronic diseases in the previous month. Exclusion criteria were cancer or cancer history and alcohol drinking. Blood was drawn between 10 a.m. and 12 p.m.

### 2.2. Isolation of PBMC

PBMCs were isolated from 15 mL of whole blood by centrifugation through Lymphoprep gradient centrifugation (Axis-Shield PoC, Norway). Briefly, blood was centrifuged at 1500 ×g for 30 min, and the mononuclear cells at the interface were carefully removed and washed three times in phosphate buffer saline (PBS).

### 2.3. Total RNA Extraction and Purification

The cell pellet was lysed in TRI-Reagent solution (Molecular Research Center, OH, USA) and immediately stored at −80°C until further processing. Total RNA extraction was done according to the manufacturer's protocol. Air-dried RNA pellet was solubilized in RNase-free water and purified using RNeasy Mini Kit columns (QIAGEN, Canada) according to the kit's manual instructions. Nucleic acid concentrations were determined at 260 nm by NanoDrop 1000 A spectrophotometer (Thermo Scientific, USA), while the RNA quality was assessed by the RNA 6000 Nano LabChip kit using the Agilent 2100 Bioanalyzer (Agilent Technologies, Germany).

### 2.4. Gene Expression Profiling

Gene expression profiling of PBMC samples from nine octo/nonagenarians and nine offspring was performed using 18 Human Gene 1.0 ST Array chips. Each chip contains 764,885 25-mer probe sets corresponding to 28,132 unique transcripts. Labelled target for the microarray experiment was prepared using 150 ng of total RNA. cDNA was synthesized using the GeneChip WT (Whole Transcript) Sense Target Labelling and Control Reagents kit as described by the manufacturer's protocol (Affymetrix, Santa Clara, USA, http://www.affymetrix.com/support/technical/manuals.affx). The sense cDNA was then fragmented by UDG (uracil DNA glycosylase) and APE 1 (apurinic/apyrimidic endonuclease 1) and biotin-labelled with TdT (terminal deoxynucleotidyl transferase) using the GeneChip WT Terminal Labelling kit (Affymetrix, Santa Clara, USA). Hybridization was performed using ~25 ng/*μ*L of biotinylated target, which was then incubated in the GeneChip 1.0 ST Array at 45°C for 17 hours. Following hybridization, nonspecifically bound material was removed by washing and detection of specifically bound target was performed using the GeneChip Hybridization, Wash and Stain kit via the GeneChip Fluidics Station 450/250 (Affymetrix, Santa Clara, USA). The arrays were scanned using the GeneChip Scanner 3000 7G (Affymetrix, Santa Clara, USA), where CEL file was produced by GeneChip operating software (GCOS) (Affymetrix). Finally, the raw data was extracted from the scanned images and analyzed with Expression Console software (Affymetrix, Santa Clara, USA). For each sample, Affymetrix default settings were used, and statistical parameters such as background, noise, and spike-in controls were within acceptable limits. All procedures were performed at UKM Medical Molecular Biology Institute (UMBI), UKM.

### 2.5. Data Analysis

Data acquisition and global background normalization were obtained using GeneSpring GX 10 software. Entities were filtered with the range of interest of 100 for upper percentile cutoff and 20 for lower percentile cutoff and retained with at least 100% of the values in any two out of two conditions within range. Genes that do not meet the criteria for differential expression were removed by computing *t*-test, followed by the Benjamini-Hochberg method for false discovery rate (FDR) control adjustment, where an FDR less than 5% was chosen with significance levels of *P* < 0.05 for the factor of age and their interaction for each gene. Annotation and ontology analyses were done using the Pathway Studio 7.0 software and Database for Annotation, Visualization and Integrated Discovery (DAVID, http://apps1.niaid.nih.gov/David/). The degree of enrichment for gene ontology and heat maps were also generated by Gene Set Enrichment Analysis (GSEA) using nonparametric Kolmogorov-Smirnov statistical test to calculate the *P* value indicating the significance of the expression changes, according to the ranking of the genes in our experimental dataset across every pathway in the database (enrichment score).

Highest fold change obtained was 2.63 and the lowest fold change was 2.47 for an unknown protein. Significant genes that changed by less than 1.2-fold with adjusted *P* > 0.05 were removed from subsequent analysis. Since the expected differences of transcriptomic expression due to aging are much smaller and difficult to detect [4], we have opted to use 1.2-fold as cutoff level as has been reported in various studies of aging [3, 7]. Hierarchical clustering was performed using differential distance metrics and centroid linkage rule of the replicates per condition. Analysis of overrepresentation of specific biological pathways by the resulting list of genes was conducted via Fisher's exact test. Pathway Studio 7.0 from Ariadne was mainly used for analysis and generating pathway figures. Functional attribution was made according to online databases such as SOURCE (http://source.stanford.edu/), GenAge (http://genomics.senescence.info/genes/) [8], and biological interpretation was derived from the literature search.

### 2.6. Real-Time RT-PCR

Real-time quantitative reverse transcription polymerase chain reaction (RT-PCR) was performed to quantitate and verify expression changes resulting from the microarray experiments. Four upregulated and eight downregulated genes were selected for validation. Genes and forward/reverse primers used for RT-PCR were as in Table 1.

The same RNA samples used in the microarray experiment were subjected to two-step RT-PCR using iScript cDNA Synthesis Kit and iQ SYBR Green Supermix (Bio-Rad Laboratories, USA). Fluorescence was measured using iCycler iQ5 Real-Time PCR Detection System (Bio-Rad Laboratories, USA). Briefly, 500 ng of total RNA was reverse-transcribed according to manufacturer's instructions. Each 20 *μ*L aliquot contained 4 *μ*L of 5x iScript reaction mix, 1 *μ*L of iScript reverse transcriptase, and 15 *μ*L of total RNA or water as negative control. The reaction mix was incubated for 5 minutes at 25°C, 30 minutes at 42°C, and 5 minutes at 85°C to obtain the cDNA template. The genes were amplified with a 25 *μ*L of reaction mix consisted of 12.5 *μ*L of iQ SYBR Green Supermix, cDNA template and primers were added at 200 nM final concentrations. Initial denaturation of DNA was carried out at 95°C for 3 minutes. Forty amplification cycles were performed, each cycle consisting of denaturation (95°C, 10 s) and annealing and extension (61°C, 30 s). The data collection and real-time analysis were performed at 95°C for 1 minute and 55°C for 1 minute, respectively. Each sample was amplified in duplicates, and the results were normalized with *β*-actin (ACTB) as reference gene. Fold change in octo/nonagenarians was determined by the Ct comparative method, using the average of Ct values after subtraction with the Ct value of ACTB from nine individuals of each octo/nonagenarians and offspring groups.

## 3. Results

### 3.1. Subject Demographics

A total of four male and fourteen female healthy octo/nonagenarians (86.1 ± 6.0 years old) and offspring (50.2 ± 4.0 years old) subjects were recruited from villages in Pahang with nine subjects in each group. No significant differences in body mass index (BMI) values were observed between the two groups (Table S1, Supplementary Material available online at http://dx.doi.org/10.1155/2013/189129). There were no significant differences in the total RNA recovered from PBMCs (data not shown); and the quality of the RNA was consistent between octo/nonagenarians and offspring with an average RNA integrity number (RIN) of 8.14 ± 0.38.

### 3.2. Gene List Selection

A total of 18 individual chips were analyzed by GeneSpring GX 10 software. *P* value computation was made with asymptotic assumptions and Benjamini-Hochberg multiple testing corrections estimates of the microarray dataset to generate a *t*-test statistic. Analysis revealed that 477 genes were significantly (*P* < 0.05) age-induced and 335 genes were significantly age-repressed with fold change ≥1.2.

The complete list of 812 differentially expressed genes is available in Table S2. At present, only selected differentially expressed genes including forkhead box O4 (*FOXO4*), TERF1 (TRF1)-interacting nuclear factor 2 (*TINF2*), X-ray repair cross-complementing protein 6 (*XRCC6*), beclin 1 (*BECN1*), and upstream transcription factor 1 (*USF1*) that have attracted our interest will be discussed. The chosen candidates were selected based on current knowledge to aging in general (e.g., oxidative stress leading to DNA damage) or to immune system, a similar effect of age on expression of genes in the same functional group and/or comparable effect of aging on immune cells gene expression in individuals.

Gene set comparison was also conducted on a list of 1312 significantly expressed genes (*P* < 0.05) with fold change >1.0 (Table S2) using the Gene Set Enrichment Analysis (GSEA) method to allow smaller degree of changes to be identified as functional category of genes (gene sets) that are regulated together. Furthermore, a computation of *P* value to determine whether the overlapping observed between the entities and the pathway is due to chance was done by Fisher's exact test.

Gene sets that may be relevant to the regulation of age-related changes between octo/nonagenarians and offspring were identified. Seven gene sets including cell growth, response to stress, response to DNA damage stimulus, chromatin modification, and phospholipid biosynthetic process were found to be downregulated in octo/nonagenarians, while 12 gene sets such as inflammatory and immune response, insulin action, apoptosis, cellular metabolic process, and cell cycle regulation were shown to be upregulated (Table 2). Fisher's exact test revealed gene ontology, transcription and insulin signalling with the most overlapping entities with 113 and 70 entities, respectively. Other gene ontologies such as translation, metabolic process, and cell cycle were overlapped with more than 30 entities. The gene ontology was ranked based on the highest *P* value (Table 3).

**Figure 1:**
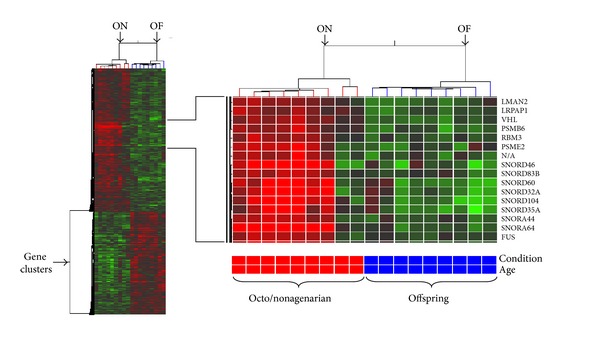
Hierarchical clustering of octo/nonagenarian (ON) versus offspring (OF). Most similar expression profiles are joined together to form a group. The expression profiles analyzed in this figure corresponded to the 812 genes that were found to be changed significantly (fold change ≥1.2; *P* < 0.05) in octo/nonagenarians. Bright red and green indicate high and low expression, respectively.

The significant up- or downregulated genes in octo/nonagenarian were reported with their *P* values and fold changes and sorted by functional group (Table 4). Some gene expression changes less than 1.2-fold (fold change ≥1.16) were worth mentioning based on the potential role in aging. Hierarchical clustering of the dataset generated by GeneSpring GX 10 software revealed further differences between the octo/nonagenarian and offspring groups datasets denoted by the letters ON and OF, respectively. The octo/nonagenarians datasets were subgrouped accordingly from the offspring microarrays (Figure 1). The global background intensity of each microarray was normalized (as described in Section 2).

### 3.3. Microarray Results Validation

In order to validate the results in Table 3, mRNA transcript levels of four upregulated and eight downregulated genes were quantified by real-time RT-PCR using the 18 PBMCs samples from each subject. The genes were selected based on a chosen list of significant biological processes generated by GSEA data, Fisher's exact test for enriched entities and pathways (Tables 2 and 3), and were ranked as overrepresented in the particular chosen pathway built from the gene set by Pathway Studio 7.0 software.

The relative differences in gene expression of four upregulated genes generated by microarray data were comparable to those of RT-PCR evaluations. For example, the expression of *FOXO4* (FC = 1.27) appeared to be upregulated in octo/nonagenarian and, similarly, it was validated by RT-PCR (FC = 1.45) in octo/nonagenarian samples. *BAK1* which represents the biological process of regulation of apoptosis, with only 1.52-fold change in the octo/nonagenarian microarray, was validated by RT-PCR with a greater fold change of 1.91 (Table 5).

Furthermore, the *HSPA8* mRNA levels in octo/nonagenarian were found to be markedly downregulated (−4.01) as assessed by RT-PCR, compared to a lesser fold change (−1.47) in the microarray analysis. Overall, the fold changes of differentially expressed genes obtained by microarray analysis (*t*-test independent, GeneSpring GX 10 software) and RT-PCR were equivalent.

### 3.4. Functional Categorization

**Figure 2:**
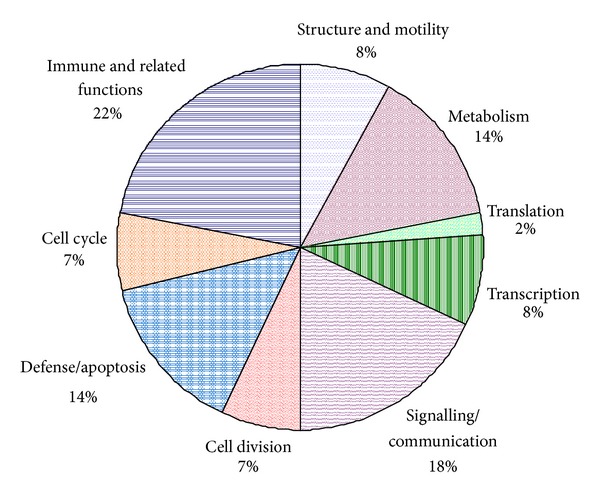
Summary of the functional attribution of significant differential expressed genes listed in Table S2, with percentage repartition of differentially regulated genes of octo/nonagenarians compared to offspring.

The functional categorizations of the chosen genes in octo/nonagenarian listed in Table S2 are summarized in Figure 2. Genes that were grouped under immune and related function (22%) and signalling and communication (18%) were shown to be most affected by age, while only 2% of the expression of genes contributing to translation changed with age. A rearrangement by functional categorization of the significant genes from the entire dataset is summarized in Table 3. Overall, the pattern of gene expression in octo/nonagenarian showed a decline in response to stress, chromatin modification, and low response to DNA damage stimulus. Upregulation of genes in octo/nonagenarian that code for cell cycle regulation, with enhanced expression of proapoptotic genes and downregulation of antiapoptotic genes, suggests increased apoptosis events in aging cells. An increase of metabolic process might indicate a possible rise in counterbalance of insulin signalling and cellular metabolic efficiency in the cells, while a decrease of positive regulation of inflammatory response may be a sign of decreased inflammatory response in octo/nonagenarian.

## 4. Discussion

### 4.1. Alterations in Gene Expression Patterns of Octo/Nonagenarians

Genes such as peroxiredoxin (*PRDX2*, *PRDX5*) or gene families such as FOXO transcription factors, insulin growth factors, autophagy-related genes beclin 1 (*BECN1*), and sirtuins (*SIRT7*) found to be differentially expressed in this study were similarly reported in an aging mouse study [9], favourable to successful aging. However, within these gene families, the upregulation of specific genes such as *FOXO4*, *SIRT7*, and *BECN1 *and downregulation of *IGF2R* were seldom reported in human aging studies. In addition, specific genes such as S100 calcium binding protein A4 and A6 (*S100A4*, *S100A6*) were also differentially expressed, in agreement with the findings of other gene expression studies involving aging cells of mice, rats, and humans [10].

Gene expression changes in aging PBMC in the current study suggested an increase in immune response and apoptosis or cell death with age which were also similarly reported in the human brain [5]. Also a decrease of cellular stress response and an increase of DNA repair mechanism (Table 2) may be critical factors that favour survival in octo/nonagenarians.

### 4.2. Functional Annotation Clustering and Genes Differentially Expressed with Age

#### 4.2.1. Immune and Related Functions

Abrupt changes in the immune system were detected in healthy individuals aged over 75 years old [2]. Among the 812 genes differentially expressed with age in octo/nonagenarian PBMC, the top cluster were the immune-related functions, followed by signalling transduction, metabolism, and apoptotic pathway (Figure 2). A study by Vo et al. [3] supported these observations where aged lymphocytes are more responsive toward apoptotic stimuli. One of the major characteristics of the aging process, called inflammaging, arises from continuous antigenic challenge. The inflammatory genes such as tumor necrosis factor superfamily member 4 (*TNFSF4*), CD40 ligand (*CD40LG), *interleukin 6 signal transducer (*IL6ST*), and cysteinyl leukotriene receptor 2 (*CYSLTR2*) were found to be downregulated in octo/nonagenarians.

A study conducted by Zuliani et al. [11] reported an increase of plasma soluble gp130 (IL6ST) levels, which plays an important role in response to environmental stress, in 997 older subjects with metabolic syndrome mediated by insulin resistance. In this context, changes in inflammation and the proinflammatory genes observed in aged subjects in this study may be the result of a favourable reaction helping older people to cope with chronic antigenic stressors. However, the association between *IL6R *genotype and longevity has not been reported despite strong evidence for association of cardiovascular disease with IL-6 and sIL-6R phenotypes [12]. In agreement with Jylhävä et al. [13], reduced expression of *CD40LG *in octo/nonagenarians (this study) which functions in full T-cell and B-cell signalling and activation suggests that the aged immune system failed to respond adequately to antigen stimuli.

Lipid mediators, cysteinyl leukotrienes (CysLTs), were downregulated in octo/nonagenarians, which are known to possess potent proinflammatory action. A decline of *CysLT(2)R* in transgenic mice overexpressed with human *CysLT(2)R* significantly reduces myocardial infarction damage [14]. It is possible that the decrease of *CysLT(2)R* offers benefit to the aged system, although the role of *CysLT(2)R* needs to be further elucidated.

#### 4.2.2. Cell Cycle and Apoptosis

The increased expression of proapoptotic genes such as BCL2-antagonist/killer 1 (*BAK1*) and TNFRSF1A-associated via death domain (*TRADD*) in aged PBMC in this study further emphasized that multiple cell types are poised for apoptosis in aging phenotype. *BAK1* is a crucial mediator of B-cell death and plays a role in the prevention of autoimmune disease. Elevated mRNA and protein expression of *TRADD* have been associated with increased apoptosis in lymphocytes from aged subjects [15]. This may imply that when encountering an oxidative challenge, PBMCs readily undergo apoptosis, whereby they more efficiently eliminate damaged cells, thus extending the life-span of the healthy cells.

#### 4.2.3. Response to Stress and Oxidative Damage

Major downregulation of genes reported in microarray studies of aged model organisms [10] and PBMC observed in current study may be linked to increased oxidative stress and damage, where over 41% of the differentially expressed genes were age-repressed in octo/nonagenarian PBMC. Also, heat shock 70 kDa protein 8 (*HSPA8*), heat shock 27 kDa protein 1 (*HSPB1*), heat shock 90 kDa protein 1, and beta (*HSP90AB1*) mRNA were found to be decreased, while heme oxygenase 2 (*HMOX2*)*, PRDX2*, *PRDX5, *and *FOXO4 *from octo/nonagenarians were found to be increased.

Aged individuals develop a constitutively low level of several chaperones including HSPA8, HSP27, and HSP90 [16]. HMOX2, PRDX2, and PRDX5 are essential enzymes associated with oxidative stress in many cell types and organisms such as human endothelial cells [17] and *Drosophila *[18]. Overexpression of *PRDX2* in *C. elegans *[19] and *PRDX5* in *Drosophila *[18] increased resistance to oxidative stress and extended their life-span. *HMOX2* plays a critical role in cell defence against oxidative stress [17], while FOXO4 transcription factor possesses antioxidative protection through a mechanism of *O*-GlcNAcylation and improves cell survival in response to oxidative stress [20]. FOXO4 is also able to modulate the expression of genes involved in oxidative stress dependent apoptosis, cell cycle arrest, DNA damage repair, and other cellular functions. Genes involved in insulin signalling including FOXO transcription factors integrate longevity pathways and metabolic signals in a complex interaction which affects the life-span determinant pathways, such as the beneficial effects of caloric restriction which may be modulated by deacetylation of FOXO4 by SIRT1 [20]. The findings that these transcripts increased in octo/nonagenarians may be explained by compensatory induction of these genes to cope with oxidative stress and damage in aging. Moreover, Terry et al. [21] suggest that low serum Hsp70 might be a marker for health given that long-lived individuals might have less cellular stress to respond to.

#### 4.2.4. DNA Repair and Telomere Maintenance

Defects in DNA repair mechanisms and telomere maintenance processes may accelerate the accumulation of oxidatively modified proteins and nucleic acids, promote the deposition of toxic protein aggregates, mitochondrial dysfunction, and aberrant gene transcription and, in turn, may lead to mutations in cancer [22]. Our results suggest an overexpression of genes crucial for genomic stability such as *XRCC6, TINF2, *TRF2-interacting telomeric protein 1 (*TERF2IP*), flap endonuclease 1 (*FEN1*), and calcium and integrin binding 1 (*CIB1*).

The ability to retain a high level of XRCC6, a DNA repair gene and part of the DNA-dependent protein kinase (DNA-PK) complex, may partially contribute to the long life-span in a longevity community in Seoul, Korea [22]. TINF2 and TERF2IP are a part of the shelterin complex which functions to prevent the activation of a DNA damage response at chromosome ends. TINF2 is essential for maintaining a functional telomere capped structure, while the role of TERF2IP is associated with telomere protection [23]. Furthermore, FEN1 is involved in regulating telomerase activity at telomeres, where mammalian cells expressing low levels of FEN1 showed increased telomere instability [24]. Finally, CIB1 is a DNA-PKcs-interacting protein and plays a positive role in telomere length maintenance [25]. Increased levels of these genes may provide proper protection, maintenance of mammalian telomeres, and DNA stability during aging. A recent study by Dekker et al. [26] reported that fibroblast strains obtained from nonagenarians of the Leiden 85-plus study with a high maximum proliferation capacity are less likely to go into stress-induced cellular senescence and have longer telomeres. On the other hand, there is a possibility that some of the age-related changes of gene expression might reflect adaptive changes; for example, the genes known to regulate specific pathways such as the DNA repair enzymes and the cell cycle regulators may function as a part of other signalling pathways [10].

#### 4.2.5. Metabolism-Related Function

Gene set for metabolism-related function including energy production by mitochondria oxidative phosphorylation (OXPHOS), insulin/IGF-1 signalling, maintenance of cholesterol levels, and lipid and lipoprotein was shown to be upregulated in octo/nonagenarian.

Age-related decline in OXPHOS enzyme activities in humans may be due to the accumulation of mutations in mtDNA. Yet, energy metabolism was shown to be generally preserved in long-lived subjects and centenarians [27]. Transcript components of OXPHOS complex I, mitochondrial respiratory chain (*NDUFV1, NDUFA13, NDUB10, and NDUFS6)*, II succinate dehydrogenase (*SDHA, SDHB*), III ubiquinol-cytochrome-c reductase (*CYC1, UQCRFS1, and UQCR*), IV cytochrome c oxidase (*COX5B, COX6A1, and COX8A*), and surfeit locus protein 1 (*SURF1*) were found to be abundant in octo/nonagenarians. Studies on long-living Ames dwarf mice showed that several components of OXPHOS system were increased relative to wild-type mice, which suggests enhanced mitochondrial function and efficiency [9]. Moreover, the expression of four genes that are involved in ATP production in complex V (ATP synthase) was altered in octo/nonagenarians where *ATP5E *and *ATP5I *were increased, while *ATP2A2 *and *ATP2CI* were decreased. Decline of complex V protein activities with age may be caused by oxidative protein modification.

It is hypothesized that the genes involved in insulin/IGF-1 signalling might also be important for human longevity, based on the reported association between decreased insulin/IGF-1 signalling and longevity in worms, flies, and mice [28]. Expression of genes involved in insulin/IGF (IIS) pathway such as insulin-like growth factor 2 receptor (*IGF2R*), phosphoinositide-3-kinase, subunit 4 (*PIK3R4*), phosphoinositide-binding protein *PIP3-E *(*IPCEF1*), protein kinase C alpha (*PRKCA*), protein kinase A beta (*PRKACB*), and phosphatidylinositol-3-phosphate 5-kinase (*PIP5K3*) was declined in octo/nonagenarians. Organisms with reduced IIS activity are resistant to a variety of cellular stresses, suggesting that mechanisms counteracting stress are enhanced [28]. Moreover, extreme human longevity seen in centenarians correlated with a low degree of insulin resistance [29]. It is expected that long-lived people are insulin sensitive all through their life-span which may protect them from age-related decline of insulin action and its associated diseases. Nevertheless, results from human studies have been inconsistent, where disruptions in insulin signalling have been linked with age-related diseases such as insulin resistance and diabetes [30].

Low-density lipoprotein related-receptor associated protein-1 (*LRPAP-1*) gene which functions as a transporter of amyloid beta protein (A*β*P) was upregulated in octo/nonagenarians. According to Zhang et al. [31], an increase of *LRPAP-1* expression resulting from supplementation of Chinese traditional medicine “Bushen Yinao Pian” on senescence-prone mouse 8/Ta (SAMP/Ta) may delay age-related cognitive defects. Finally, downregulation of stearoyl-CoA desaturase (*SCD1*) was observed in octo/nonagenarians. SCD1 catalyses the biosynthesis of mono-unsaturated fatty acids from palmitoyl-CoA and stearoyl-CoA, reported to be important in the maintenance of phospholipid membrane fluidity for normal cellular function. Decreasing SCD1 expression might protect against obesity and insulin resistance, where mice deficient in SCD1 are resistant to metabolic syndrome and are insulin sensitive [32].

#### 4.2.6. Autophagy and Vesicular Trafficking

Autophagy functions in housekeeping and quality control that contribute to health and longevity. In some cases, autophagy can protect cells against intracellular pathogens. Beclin 1 (*BECN1*), the central protein regulator of autophagy, was overexpressed in octo/nonagenarians. Deletion of *bec-1 *inverts longevity in worms and a recent study demonstrated *Becn1* as potential therapeutic target in Alzheimer's disease, where lack of Becn1 modulates amyloid precursor protein metabolism and promotes neurodegeneration in mice [8].

#### 4.2.7. Transcription and Protein Synthesis

Increased mRNA abundance of *SIRT7, USF1, NRF, *and *KEAP1* was shown in octo/nonagenarian group. According to Ford et al. [33], SIRT7 is a positive regulator of RNA polymerase I transcription and is essential for cell survival in mammals. USF1 regulates genes involved in inflammation, lipid, and glucose metabolism. A study by [34] reported the association of USF1 haplotype with lower cholesterol levels and decreased risk of early-onset coronary atherosclerosis in young adults and suggests the involvement of USF1 in the regulation of human longevity. Nuclear respiratory factor 1 (*NRF1*), a DNA-binding transcription factor, plays a role in nuclear-mitochondrial interactions and other cellular functions such as protein synthesis, DNA repair, and cell proliferation. As *NRF1* gene knockout mice results in mtDNA instability and embryonic lethality, thus, regulation of *NRF1* is crucial in order to balance the cell energy demands [35]. Besides acting as a repressor protein that binds NRF2 to promote its proteasomal degradation, kelch-like ECH-associated protein 1 (KEAP1) also plays a central role in regulating the protective response [36]. Perhaps the induction of transcription in older cells relative to younger cells indicates that the aging profile shows an active response to damage and genome instability.

## 5. Conclusion

The current findings suggest that long-lived phenotype has cellular survival mechanisms that may guard against oxidative stress and DNA damage by activating gene sets common to DNA repair, telomere maintenance, antioxidant response, autophagy pathway, and metabolism-related genes. This implies that a boost of key DNA repair elements and efficient cellular trafficking may promote lower ROS production or oxidative substrate damage [18, 22]. Importantly, the unique regulation of possible “innate” genes in octo/nonagenarians' PBMC may shed some light on the pathophysiology of the long-lived phenotype. This study has also revealed potential candidate genes such as *XRCC6, FOXO4, IGF2R, SIRT7,* and *KEAP1* which may impact the aging process and survival and henceforth contribute to human longevity.

## Supplementary Material

Table S1: Body mass index (BMI) values, age and gender of octo/nonagenarian and offspring groups. The P-value refers to a two-tailed t-test for the differences in means between the BMI values in octo/nonagenarians vs. offspring. A total of four male and 14 female subjects were recruited in this study.
Table S2: A complete list of 812 genes which were differentially expressed with significant levels of P<0.05, Benjamini-Hochberg false discovery rate (FDR) adjustment and fold changes ≥1.2. The fold-change refers to the ratio of the expression values of octo/nonagenarians over offspring.
Table S3: A complete list of 1312 genes which were differentially expressed with significant levels of P<0.05, Benjamini-Hochberg false discovery rate (FDR) adjustment and fold changes >1.0. The fold-change refers to the ratio of the expression values of octo/nonagenarians over offspring.

## Figures and Tables

**Table 1:** 

Accession number	Gene symbol	Forward (5′-3′)	Reverse (5′-3′)	Product size (bp)
NM_005938	*FOXO4 *	ccagcttcagtcagcagttatg	agagaccactccgagatagcag	118
NM_153201	*HSPA8 *	aggcccaaggtccaagtaga	agcatctttggtagcctgacg	186
NM_000074	*CD40LG *	agccagcctctgcctaaagt	gctcacttggcttggatcag	175
NM_003672	*CDC14A *	agcccgtttaccaacctctt	tggtgtgctcctctgtcttg	157
NM_001469	*XRCC6 *	aagccgttggtactgctgaag	ctccagacacttgatgagcagag	123
NM_003326	*TNFSF4 *	gctacttctcccaggaagtcaac	gtaagtcagagaggccaccatc	116
NM_006311	*NCOR1 *	agcggctatgctctctaccag	gctgaaggacttcccactctc	163
NM_002737	*PRKCA *	aggatgatgacgtggagtgc	gacgaagtacagccgatcca	119
NM_004064	*CDKN1B *	tccggctaactctgaggacac	caggtcgcttccttattcctg	104
NM_032983	*CASP2 *	agtggtgctagccaaacagc	aggcagcaagttgaggagttc	145
NM_001681	*ATP2A2 *	ctacctcatctcgtccaacgtc	tcaccagattgacccagagc	110
NM_001188	*BAK1 *	cggcagagaatgcctatgag	agtcaggccatgctggtaga	137
NM_001101	*ACTB *	cctggacttcgagcaagagat	aggaaggaaggctggaagagt	141

**Table 2:** A list of statistically significant categories in octo/nonagenarians based on gene set enrichment analysis (GSEA) sorted according to the normalized enrichment score (NES).

Biological process (GSEA)	Normalized enrichment score (NES)^a^	Median change	*P* value^b^
Inflammatory response	2.07	1.01	0.00*E* + 00
Immune response	1.93	1.02	0.00*E* + 00
Insulin action	1.57	1.02	0.00*E* + 00
Regulation of lipid metabolic process	1.50	1.02	3.80*E* − 02
Cholesterol biosynthetic process	1.46	1.04	2.10*E* − 02
Induction of apoptosis	1.42	1.02	0.00*E* + 00
Cellular metabolic process	1.41	1.02	3.80*E* − 02
Antiapoptosis	1.31	1.01	3.40*E* − 02
Cell cycle regulation	1.30	1.01	0.00*E* + 00
Cell proliferation	1.28	1.00	0.00*E* + 00
Cell growth	−1.42	−1.03	2.40*E* − 02
Response to stress	−1.46	−1.01	0.00*E* + 00
Response to DNA damage stimulus	−1.49	−1.08	3.80*E* − 02
Chromatin modification	−1.52	−1.02	0.00*E* + 00
Positive regulation of cell proliferation	−1.63	1.01	0.00*E* + 00
Phospholipid biosynthetic process	−1.56	−1.04	0.00*E* + 00
Positive regulation of inflammatory response	−1.75	−1.03	0.00*E* + 00

^a^Positive NES indicates an upregulation in octo/nonagenarians, whereas negative NES reflects an upregulation in offspring samples.

^b^ *P* value estimates the statistical significance of the enrichment score for a single gene set using Kolmogorov-Smirnov statistical test.

**Table 3:** Analysis of gene list (*P* < 0.05, FDR) by Fisher's exact test.

Biological process	Source^a^	List hits^b^	Population hits^c^	*P* value^d^
Transcription	GO:0006355	113	2246	5.63*E* − 13
Translation	GO:0006412	40	635	1.08*E* − 07
Insulin action	GO:0046626	70	905	2.45*E* − 05
Cell cycle	GO:0007049	30	539	4.43*E* − 05
Cell division	GO:0051301	18	266	1.42*E* − 04
Response to stress	GO:0006950	16	239	3.67*E* − 04
Double-strand break repair	GO:0006302	5	30	8.14*E* − 04
Cell cycle arrest	GO:0007050	5	115	1.66*E* − 03
Regulation of apoptosis	GO:0006915	12	177	1.72*E* − 03
Chromatin modification	GO:0016568	12	186	2.60*E* − 03
Response to DNA damage stimulus	GO:0006974	14	236	2.60*E* − 03
Metabolic process	GO:0008152	35	858	3.38*E* − 03
Induction of apoptosis	GO:0006917	12	207	6.10*E* − 03
Immune response	GO:0006955	18	604	2.55*E* − 01
Inflammatory response	GO:0006954	10	293	2.00*E* − 01

^a^Source is from the Gene Ontology, http://www.geneontology.org/.

^b^Number of genes present in this set of 812 genes; *P* < 0.05, FDR (list hits) (see Supplementary Table S2).

^c^Number of genes in each category present in the entire array (population hits).

^d^The *P* value refers to Fisher's exact test (see experimental procedures).

**Table 4:** Genes differentially expressed in PBMC of octo/nonagenarians^a^ (*n* = 9) versus offspring (*n* = 9). Statistical analysis was carried out with *t*-test. Genes were classified based on functional category.

Function	Gene symbol	Corrected *P* value^b^	*P* value^c^	Fold change^d^	Regulation	Gene name	Entrez ID^e^
Cell cycle	*BAK1 *	3.88*E* − 04	1.25*E* − 07	1.52	Up	BCL2-antagonist/killer 1	NM_001188
	*CDKN1B *	4.92*E* − 03	2.94*E* − 05	1.43	Up	Cyclin-dependent kinase inhibitor 1B (p27, Kip1)	NM_004064
	*CDK3 *	2.38*E* − 03	3.62*E* − 06	1.31	Up	Cyclin-dependent kinase 3	NM_001113324
	*TRADD *	4.60*E* − 03	2.28*E* − 05	1.48	Up	TNFRSF1A-associated via death domain	NM_003789
	*CASP2 *	3.17*E* − 02	1.35*E* − 03	1.33	Down	Caspase 2, apoptosis-related cysteine peptidase	NM_032982
	*CDC14A *	4.41*E* − 03	2.16*E* − 05	1.53	Down	CDC14 cell division cycle 14 homolog A (*S. cerevisiae*)	NM_003672

Immune and related function	*CD40LG *	1.09*E* − 02	1.81*E* − 04	1.46	Down	CD40 ligand (TNF superfamily, member 5, hyper-I gM syndrome)	NM_000074
	*CYSLTR2 *	9.86*E* − 03	1.43*E* − 04	1.62	Down	Cysteinyl leukotriene receptor 2	NM_020377
	*IL6ST *	1.31*E* − 02	2.64*E* − 04	1.46	Down	Interleukin 6 signal transducer (gp130, oncostatin M receptor)	NM_002184
	*TNFSF4 *	4.99*E* − 02	3.02*E* − 03	1.93	Down	Tumor necrosis factor (ligand) Superfamily, member 4 (tax-transcriptionally activated glycoprotein 1, 34 kDa)	NM_003326

Metabolism							
	*ATP5E *	3.78*E* − 02	1.88*E* − 03	1.30	Up	ATP synthase, H^+^ transporting, mitochondrial F1 complex, epsilon subunit	NM_001001977
*Oxidative phosphorylation *	*ATP5I *	1.39*E* − 02	2.88*E* − 04	1.26	Up	ATP synthase, H^+^ transporting, mitochondrial F0 complex, subunit E	NM_007100
	*COX5B *	6.46*E* − 03	6.16*E* − 05	1.24	Up	Cytochrome c oxidase subunit Vb	NM_001862
	*COX6A1 *	9.84*E* − 03	1.41*E* − 04	1.47	Up	Cytochrome c oxidase subunit VIa polypeptide 1	NM_004373
	*CYC1 *	4.91*E* − 02	2.92*E* − 03	1.19	Up	Cytochrome c-1	NM_001916
	*NDUFA13 *	3.31*E* − 02	1.49*E* − 03	1.16	Up	NADH dehydrogenase (ubiquinone) 1 alpha subcomplex, 13	NM_015965
	*NDUFB10 *	3.34*E* − 02	1.51*E* − 03	1.16	Up	NADH dehydrogenase (ubiquinone) 1 beta subcomplex, 10, 22 kDa	NM_004548
	*NDUFS6 *	2.75*E* − 02	1.01*E* − 03	1.21	Up	NADH dehydrogenase (ubiquinone) Fe-S protein 6, 13 kDa (NADH-coenzyme Q reductase)	NM_004553
	*NDUFV1 *	9.27*E* − 03	1.28*E* − 04	1.16	Up	NADH dehydrogenase (ubiquinone) flavoprotein 1, 51 kDa	NM_007103
	*SDHA *	4.92*E* − 03	2.95*E* − 05	1.21	Up	Succinate dehydrogenase complex, subunit A, flavoprotein (Fp)	NM_004168
	*SDHB *	4.92*E* − 02	2.94*E* − 03	1.19	Up	Succinate dehydrogenase complex, subunit B, iron sulfur [6]	NM_003000
	*UQCR *	7.01*E* − 03	7.07*E* − 05	1.21	Up	Ubiquinol-cytochrome c reductase, 6.4 kDa subunit	NM_006830
	*UQCRFS1 *	3.01*E* − 02	1.24*E* − 03	1.39	Up	Ubiquinol-cytochrome c reductase, Rieske iron-sulfur polypeptide 1	NM_006003
	*ATP2A2 *	1.31*E* − 02	2.64*E* − 04	1.37	Down	ATPase, Ca^++^ transporting, cardiac muscle, slow twitch 2	NM_170665
	*ATP2C1 *	1.07*E* − 02	1.74*E* − 04	1.22	Down	ATPase, Ca^++^ transporting, type 2C, member 1	NM_014382
	*COX2 *	4.29*E* − 02	2.32*E* − 03	1.19	Down	Cytochrome c oxidase II	NC_001807
	*ISCA1 *	2.78*E* − 02	1.05*E* − 03	1.21	Down	Iron-sulfur cluster assembly 1 homolog (*S. cerevisiae*)	NM_030940
*Insulin action *	*IGF2R *	4.85*E* − 02	2.85*E* − 03	1.28	Down	Insulin-like growth factor 2 receptor	NM_000876
	*PIK3R4 *	4.26*E* − 02	2.30*E* − 03	1.21	Down	phosphoinositide-3-kinase, regulatory subunit 4	NM_014602
	*PIP3-E *	2.01*E* − 02	5.62*E* − 04	1.34	Down		NM_015553
	*PIP5K3 *	4.54*E* − 02	2.52*E* − 03	1.17	Down	Phosphatidylinositol-3-phosphate/phosphatidylinositol 5-kinase, type III	NM_015040
	*PRKACB *	4.35*E* − 02	2.36*E* − 03	1.44	Down	Protein kinase, cAMP-dependent, catalytic, beta	NM_182948
	*PRKCA *	2.76*E* − 02	1.02*E* − 03	1.38	Down	Protein kinase C, alpha	NM_002737
*Cholesterol, lipid and, lipoprotein maintenance *	*LRPAP1 *	3.04*E* − 03	6.72*E* − 06	1.47	Up	Low density lipoprotein receptor-related protein associated protein 1	NM_002337
	*SCD *	1.23*E* − 02	2.34*E* − 04	1.23	Down	Stearoyl-CoA desaturase (delta-9-desaturase)	NM_005063

Response to stress	*FOXO4 *	3.07*E* − 02	1.28*E* − 03	1.27	Up	Forkhead box O4	NM_005938
	*HMOX2 *	4.07*E* − 02	2.13*E* − 03	1.19	Up	Heme oxygenase (decycling) 2	NM_002134
	*PIN1 *	1.04*E* − 02	1.63*E* − 04	1.24	Up	Peptidylprolyl cis/trans isomerase, NIMA-interacting 1	NM_006221
	*PRDX2 *	1.74*E* − 02	4.36*E* − 04	1.15	Up	Peroxiredoxin 2	NM_005809
	*PRDX5 *	3.17*E* − 02	1.35*E* − 03	1.27	Up	Peroxiredoxin 5	NM_012094
	*HSPA8 *	5.58*E* − 03	4.12*E* − 05	1.47	Down	Heat shock 70 kDa protein 8	NM_006597

DNA repair and telomere maintenance	*CIB1 *	3.34*E* − 02	1.51*E* − 03	1.20	Up	Calcium and integrin binding 1 (calmyrin)	NM_006384
	*FEN1 *	2.05*E* − 02	6.00*E* − 04	1.24	Up	Flap structure-specific endonuclease 1	NM_004111
	*TERF2IP *	1.46*E* − 02	3.15*E* − 04	1.16	Up	Telomeric repeat binding factor 2, interacting protein	NM_018975
	*TINF2 *	2.73*E* − 03	4.60*E* − 06	1.31	Up	TERF1 (TRF1)-interacting nuclear factor 2	NM_012461
	*XRCC6 *	2.26*E* − 02	7.02*E* − 04	1.27	Up	X-ray repair complementing defective repair in Chinese hamster cells 6 (Ku autoantigen, 70 kDa)	NM_001469
	*DICER1 *	3.45*E* − 02	1.60*E* − 03	1.28	Down	Dicer 1, ribonuclease type III	NM_177438

Translation and transcription	*USF1 *	5.58*E* − 03	3.91*E* − 05	1.33	Up	Upstream transcription factor 1	NM_007122
	*KEAP1 *	1.18*E* − 02	2.15*E* − 04	1.23	Up	Kelch-like ECH-associated protein 1	NM_203500
	*NRF1 *	4.87*E* − 02	2.88*E* − 03	1.19	Up	Nuclear respiratory factor 1	NM_005011
	*SIRT7 *	3.55*E* − 02	1.67*E* − 03	1.18	Up	Sirtuin (silent mating type information regulation 2 homolog) 7 (*S. cerevisiae*)	NM_016538

Autophagy	*BECN1 *	4.78*E* − 03	2.40*E* − 05	1.32	Up	Beclin 1, autophagy related	NM_003766
	*VPS18 *	1.81*E* − 02	4.74*E* − 04	1.26	Up	Vacuolar protein sorting 18 homolog (*S. cerevisiae*)	NM_020857

^a^The genes shown in this table are ordered according to the biological processes. For a complete list of all 812 genes significantly affected in octo/nonagenarians, see Table S2.

^b^Corrected *P* value refers to *P* value after multiple testing corrections by Benjamini-Hochberg method.

^c^ *P* value refers to *P* value before multiple testing corrections by Benjamini-Hochberg method.

^d^The fold change refers to the ratio of the expression values of octo/nonagenarians over offspring.

^e^Source of entrez ID is from National Center for Biotechnology Information (NCBI), http://www.ncbi.nlm.nih.gov/gene/.

**Table 5:** Genes differentially expressed in PBMC of octo/nonagenarian versus offspring. Statistical analysis was carried out using independent *t*-test (RT-PCR) with a Benjamini-Hochberg false-discovery rate (microarray).

Biological process	Gene symbol	Gene name	RT-PCR		Microarray data	
			Fold change	*P* value^a^	Fold change	Corrected *P* value
Cell cycle	*CDKN1B *	Cyclin-dependent kinase inhibitor 1B (p27, Kip1)	1.48	9.46*E* − 03	1.43	4.90*E* − 03
Double-strand break repair	*XRCC6 *	X-ray repair cross-complementing protein 6 (Ku autoantigen, 70 kDa)	1.49	1.34*E* − 02	1.27	2.30*E* − 02
Cell cycle arrest	*FOXO4 *	Forkhead box O4	1.45	3.49*E* − 03	1.27	3.00*E* − 02
Regulation of apoptosis	*BAK1 *	BCL2-antagonist/killer 1	1.91	3.80*E* − 02	1.52	3.90*E* − 04
Response to stress	*HSPA8 *	Heat shock 70 kDa protein 8	−4.01	1.30*E* − 04	−1.47	5.60*E* − 03
Immune response	*CD40LG *	CD40 ligand	−2.71	1.32*E* − 04	−1.46	1.10*E* − 02
Cell division	*CDC14A *	CDC14 cell division cycle 14 homolog A	−2.04	4.21*E* − 04	−1.53	4.40*E* − 03
Inflammatory response	*TNFSF4 *	Tumor necrosis factor (ligand) superfamily, member 4	−2.57	4.02*E* − 05	−1.93	4.90*E* − 02
Chromatin modification	*NCOR1 *	Nuclear receptor corepressor 1	−1.69	7.86*E* − 03	−1.43	4.40*E* − 02
Insulin action	*PRKCA *	Protein kinase C, alpha	−1.64	3.07*E* − 02	−1.38	2.70*E* − 02
Induction of apoptosis	*CASP2 *	Caspase 2	−2.08	3.66*E* − 03	−1.33	3.10*E* − 02
Metabolic process	*ATP2A2 *	ATPase, Ca^++^ transporting	−1.61	5.49*E* − 03	−1.37	1.30*E* − 02
Housekeeping gene (cytoskeleton)	*ACTB *	Beta Actin				

^a^The *P* values for the real-time reverse transcription PCR (RT-PCR) refer to a two-tailed *t*-test for the differences in means between the normalized Ct values in octo/nonagenarians versus offspring.
